# Case report: Environmental adjustment for visual hallucinations in dementia with Lewy bodies based on photo assessment of the living environment

**DOI:** 10.3389/fpsyt.2024.1283156

**Published:** 2024-03-15

**Authors:** Daiki Ishimaru, Hideki Kanemoto, Maki Hotta, Yuma Nagata, Fuyuki Koizumi, Yuto Satake, Daiki Taomoto, Manabu Ikeda

**Affiliations:** ^1^ Department of Medical Technology, Osaka University Hospital, Suita, Japan; ^2^ Department of Psychiatry, Osaka University Graduate School of Medicine, Suita, Japan

**Keywords:** DLB, visual hallucination, environmental factor, non-pharmacology, case report

## Abstract

**Background:**

Visual hallucinations (VH) are associated with visual prediction error in patients with dementia with Lewy bodies (DLB). Given this relationship, environmental adjustments have been suggested, but detailed contents for implementing such environmental adjustments and assessments are poorly documented. This case report preliminarily demonstrates methods for improving VH through our experience with two patients with DLB. We conducted familial interviews to assess the phenomenological features of VH and reviewed photographs of patients’ homes to identify the environmental triggers of VH, known as photo assessment of the living environment (PA-LE).

**Case description:**

Patient 1 was a 78-year-old woman with a Mini-Mental State Examination (MMSE) score of 11/30. She experienced seeing a stranger, children, and cats at home, which frightened her. VH frequently occurred in the living room and bedroom. The PA-LE showed that several environmental features, such as cushions on a sofa, the pattern on a carpet under a table, and clothing on hangers, were suggestive triggers of VH. Patient 2 was an 88-year-old woman with a MMSE score of 5/30. She had seen strangers, children, and animals at home, some of which were linked to a theft delusion. VH frequently occurred in the living room and bedroom. The PA-LE found that several environmental features, such as clothing on hangers and dolls, were suggestive of VH triggers. Non-pharmacological approaches were tailored to the patients’ environmental and psychological states using interviews and PA-LE. This included removing environmental triggers, reducing negative mood, and providing coping strategies for VH. This improved their VH and their caregivers’ knowledge of VH.

**Conclusion:**

Phenomenological assessments using photographs of the patient’s home could identify the environmental triggers associated with VH in patients with DLB and assist in environmental adjustments.

## 1 Introduction

Dementia with Lewy bodies (DLB) is characterized by diverse and complex clinical features such as cognitive impairment, fluctuating cognition, spontaneous Parkinsonism, rapid eye movement sleep behavior disorder (RBD), and visual hallucinations (VH). Among these clinical features, VH is frequently prevalent (1), and contributes to several negative outcomes, including delusions and caregiver burden (2, 3).

Patients with DLB tend to exhibit recurrent and complex VH features (4). Several different models have been used to understand VH through various points of view. Visual prediction error, one of these underlying mechanisms, is thought to be interactivity associated with VH (5). A relevant previous study has shown that the patients with DLB with frequent VH saw visual stimuli as meaningful illusory images in even if they were meaningless (6).

Avoiding specific triggers and settings that increase the risks of VH is suggested as a non-pharmacological management of VH (7). Given the relationship between visual prediction errors and VH, several studies have recently sought to provide an outline of environmental adjustments, such as removing trigger stimuli or increasing indoor brightness (8, 9). However, details of the content or procedure for implementing such environmental adjustments are poorly documented.

This preliminary case report describes the methods of environmental assessment and adjustment for improving VH through our experience with two patients with DLB, based on the hypothesis that VH could be partially associated with visual illusions.

## 2 Case presentation

Personal information was anonymized to protect patient privacy. Written informed consent was obtained for publication of this case report from the patients and their families.

### 2.1 Photo assessment of the living environment and assessment of the visual hallucinations.

We conducted a photo assessment of the living environment (PA-LE) (10) to identify environmental triggers of VH. The patients and their families were asked to photograph places or objects in their homes according to the prepared guidance (**Supplementary Figure 1**).

Familial interviews were conducted to assess the phenomenological features of VH with an interviewer observing a photograph of the patient’s home. This was an interactive process. The environmental triggers that might affect the occurrence of VH were identified by the multidisciplinary team that consisted of occupational therapists, psychiatrists, nurses, or psychologists based on those assessments.

As it is difficult to clearly distinguish between VH and visual illusions in clinical practice, we mainly describe the VH-like experience as VH.

### 2.2 Patient 1

A 78-year-old woman presented to our psychiatric outpatient clinic. She lived with her husband, and most of the housework and care were assisted by him.

**Figure 1:**
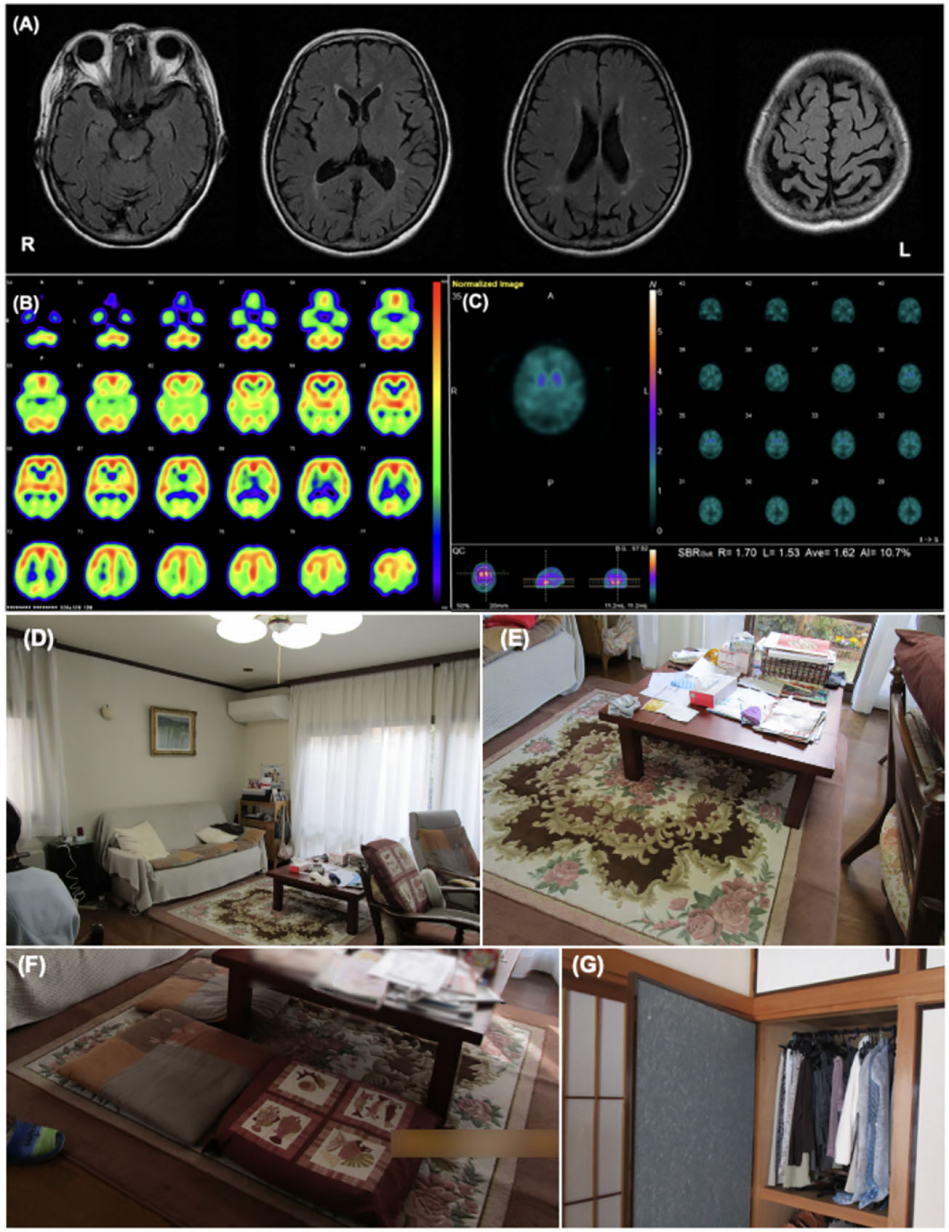
Patient 1 images **(A)** The cranial MR T2 FLAIR image shows no obvious abnormal findings except for mild atrophy of the bilateral parietal lobes. **(B)** I-^123^IMP SPECT shows reduced cerebral perfusion in the bilateral parietal, temporal, and occipital lobe while perfusion in the bilateral posterior cingulate cortex was relatively preserved. **(C)** I-^123^FP-CIT SPECT showed a decrease in accumulation of dopamine in the striatum. **(D, E)** These images show the environmental triggers associated with the visual hallucinations. Cushions on a sofa in the living room **(D)**, the pattern on a carpet under the table in the living room **(E)** were suspected of inducing her visual hallucinations as follows: “Boy sitting on the sofa” and “Child playing under the table,” respectively. **(F, G)** These images show the living environment post-intervention. The carpet pattern was hidden by a tablecloth **(F)**, and the clothes on hangers in the bedroom were stored in a closet **(G)**.

She first presented with mild amnesia and depression at the age of 70, while also exhibiting diplopia. Two years after onset, she was referred to our hospital for further examination and treatment. She exhibited mild cognitive impairment (Mini-Mental Examination State: MMSE score, 26/30) and mild Parkinsonism. She also complained of VH, in which objects moved like bugs. She occasionally had difficulty performing daily activities, indicating cognitive fluctuations. RBD did not occur. Cranial magnetic resonance (MR) imaging showed no obvious abnormalities except for mild atrophy of the bilateral parietal lobes (**Figure 1A**). ^123^I-IMP single-photon emission computed tomography (SPECT) revealed reduced cerebral perfusion in the bilateral parietal, temporal, and occipital lobes, while perfusion in the bilateral posterior cingulate cortex was relatively preserved (**Figure 1B**). ^123^I-FP-CIT SPECT revealed reduced accumulation in the right-dominant bilateral posterior striatum (**Figure 1C**). Based on these results, the patient was diagnosed with mild-stage probable DLB according to the 2005 version of the International Diagnostic Criteria by McKeith et al. (11) Eight years after onset, VH frequently occurred with fear, although it had responded to donepezil hydrochloride for a few years. A non-pharmacological approach was planned to improve her VH and accompanying fear. For pharmacological intervention, levodopa (300 mg/day), zonisamide (25 mg/day), donepezil hydrochloride (10 mg/day), eszopiclone (1 mg/day), yokukan-san (25 g/day), and mosapride citrate hydrate (15 mg/day) were prescribed.

**Table 1** shows the clinical features of Patient 1. Before the non-pharmacological intervention, the Clinical Dementia Rating (CDR) score was 2. The MMSE and Addenbrooke Cognitive Examination III (ACE-III) scores were 11/30 and 22/100, respectively, indicating general cognitive impairment. The Short Fluctuations Questionnaire (SFQ) score was 7/8, indicating cognitive fluctuations. The Movement Disorder Society Unified Parkinson’s Disease Rating Scale Part III (MDS-UPDRS Part III) score was 48/136, with rigidity in the bilateral upper limbs and trunk, bradykinesia, postural instability, and postural tremor. The total Neuropsychiatry Inventory-12 (NPI-12) score was 34/144, and the hallucination frequency and severity scores were 4/4 and 1/3, respectively. Caregiver distress regarding VH in the NPI-12 group was mild. The Zarit Burden Inventory (ZBI) score was 16/88.

**Table 1:** Clinical characteristics of Patient 1 at baseline and at 3-month follow up.

	Baseline			3 months follow-up		
CDR	2			–		
MMSE	11/30			8/30		
ACE-III	22/100			–		
SFQ	7/8			–		
MDS-UPDRS partIII	48/136			–		
PSMS	2/6			1/6		
NPI-12 score	Total score 34/144			Total score 38/144		
	Del 3/12	Hal 4/12	Agit 3/12	Del 3/12	Hal 3/12	Agit 6/12
	Dep 1/12	Anx 3/12	Eup −	Dep −	Anx 4/12	Eup −
	Ap 8/12	DI −	Irrit −	Ap 8/12	DI 1/12	Irrit −
	MD −	NB 8/12	EA 4/12	MD 1/12	NB 12/12	EA −
ZBI	16/88			20/88		
Visual hallucination						
Caregiver burden score (NPI-12 hallucination)	2/5 (mild)			1/5 (minimal)		
Frequency	Multiple times daily			A few times a week		
What the patient saw	Someone unidentifiable such as children or strangers			Someone unidentifiable such as children or visitors		
When the symptoms occurred	Evening to night-time			Evening to night-time		
Visual features associated with hallucinations	Cushions on a sofa in the living room (**Figure 1D**) Carpet pattern under the table in the living room (**Figure E**) Clothing on hangers in the bedroom (**invisible from the PA-LE**)			Cushions on a sofa in the living room Shadow of handrail on a wall in the living room		
How the patient reacted to the hallucinations	Doing nothing while being afraid of them			Touching something she saw by herself		

CDR, clinical dementia rating; MMSE, Mini-Mental State Examination; ACE-III, Addenbrooke’s Cognitive Examination III; SFQ, Short Fluctuations Questionnaire; MDS-UPDRS Part III, Movement Disorder Society Unified Parkinson’s Disease Rating Scale Part III; PSMS, Physical Self-Maintenance Scale; NPI-12, Neuropsychiatry Inventory-12; Del, delusions; Hal, hallucinations; Agit, agitation/aggression; Dep, depression/dysphoria; Anx, anxiety; Eup, elation/euphoria; Ap, apathy/indifference; DI, disinhibition; Irrit, irritability; MD, motor disturbance: NB, night-time behaviors; EA, appetite and eating abnormalities; ZBI, Zarit Burden Inventory.

VH features were assessed using interviews and the PA-LE (**Table 1**). Familial interviews revealed that she had frequently seen someone unidentifiable and a cat in their home, which frightened her. She experienced VH many times a day, mainly in the evening and at night. VH frequently occurred in living the room and bedroom. Based on both family interviews and PA-LE, we assessed whether her VH experience had any possible environmental triggers (**Figures 1D, E**). For instance, cushions on a sofa in the living room, a pattern on a carpet under the table in the living room, and clothing on hangers in the bedroom (not visible in the PA-LE) were associated with instances of VH (“Boy sitting on the sofa,” “Child playing under the table,” and “Someone is in my bedroom,” respectively), although the VH did not occur every time she saw the triggers. The patient and her husband did not know how to manage the VH.

We recommended several instructions for the management of VH (**Supplementary Table**). First, we provided the patient and her husband with suggestions of environmental adjustments, for example, to put away the cushions, hide pattern on the carpet under the table, and fold the hanging clothes, which were considered environmental triggers of VH. Second, we encouraged them to use a day care service and to spend time together in the evening and at night to reduce general anxiety. Finally, as a coping strategy we suggested that she try to touch the VH, and had caregivers respond to her to provide a feeling of security if VH occurred. There was no change in the prescribed medicine before and after the non-pharmacological intervention.

The patient had an uneventful course during the three months of follow-up. Her husband implemented the guidance provided, and the environmental triggers were reduced (**Figures 1F, G**). She began to use daycare services twice a week after receiving care instruction. The frequency score for NPI hallucinations decreased, while the scores for agitation/aggression and nighttime behaviors worsened (**Table 1**). The patient and her husband dealt with them by touching something she saw, even if a VH occurred. These changes alleviated her fear of VH, and several other aspects also changed. For example, the VH of strangers were converted into visitors. Additionally, the caregiver distress for VH decreased to a minimal level, and the husbands’ knowledge of VH was altered as follows: “She may be misunderstanding things.” However, the general caregiver burden assessed using the ZBI increased post-intervention.

### 2.3 Patient 2

An 89-year-old woman was admitted to our psychiatric ward for the treatment of hallucinations and delusions. She lived alone with her daughter, and home visiting nurses assisted most of the housework and care.

She first presented with amnesia at the age of 88. One year after onset, her memory impairment progressed, and she complained of a delusion of theft in which strangers intruded on her house and stole money. In addition, she had a systematized belief that one of them had a romantic relationship with her daughter, and she identified her daughter as one of the persecutors. She complained of VH, and that a stranger was asleep in the bed; the timeline of the delusions and VH was unclear. There was also a VH of cats that she fed in front of the house. In the same year, she was referred to our outpatient clinic and scheduled for hospitalization. Her MMSE score was 13/30 at the first visit. She sometimes had difficulty performing self-care tasks, such as using the toilet or changing clothes, indicating the presence of cognitive fluctuation. She did not show Parkinsonism and RBD. Risperidone (1.0 mg/day) and suvorexant (10 mg/day) had been administered at a previous hospital.

**Figure 2:**
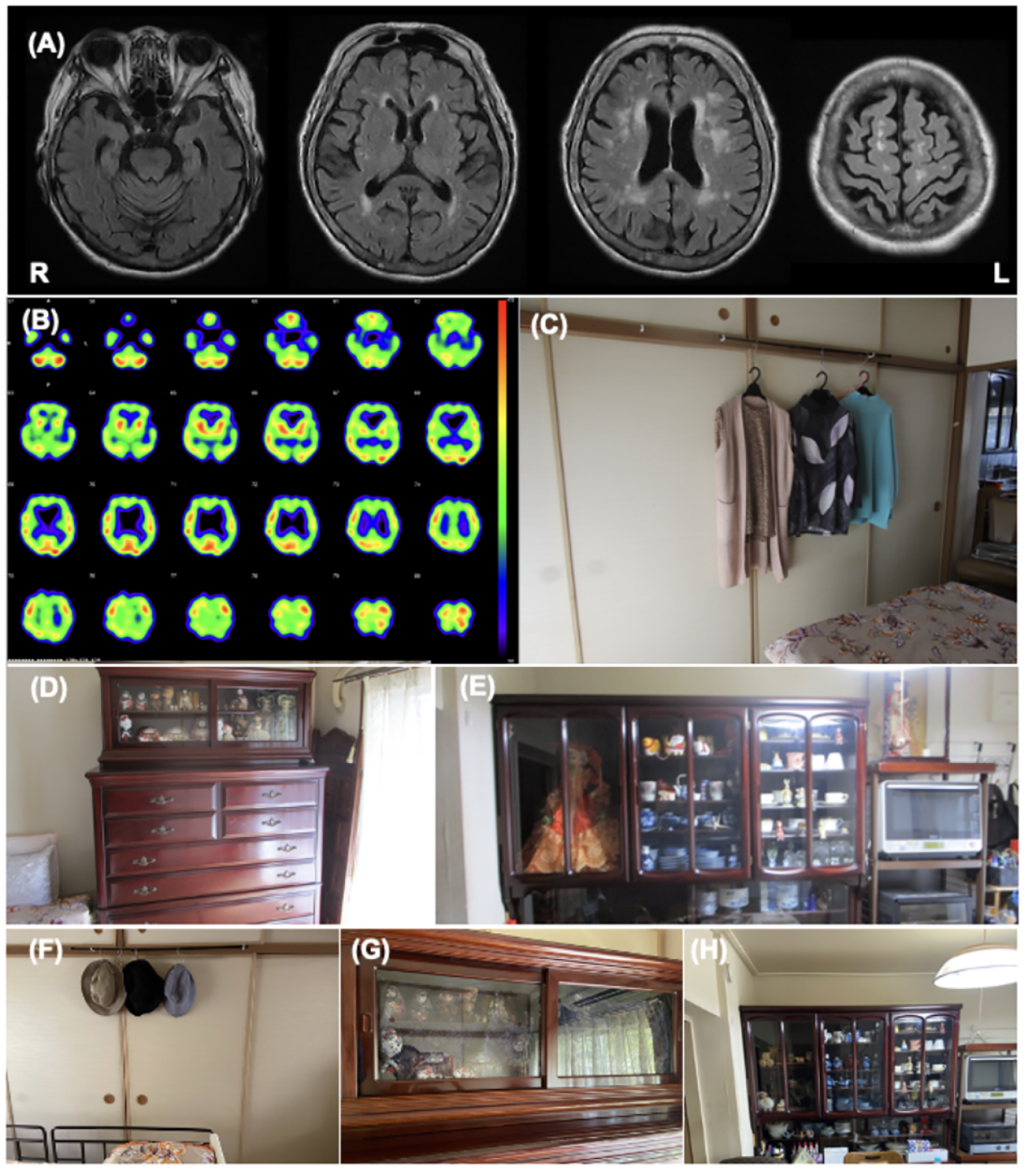
Patient 2 images **(A)** The cranial MR T2 FLAIR image shows bilateral atrophy of the medial temporal lobe with an extensive high intensity area. **(B)** I-^123^IMP SPECT shows diffuse reduction of brain perfusion. **(C-E)** These images show the environmental triggers associated with the visual hallucinations. Clothing on hangers in the bedroom **(C)**, dolls in the bedroom **(D)**, and dolls in the living room **(E)** were suspected of inducing her visual hallucinations as follows: “Male strangers intruded my home from the balcony”, or “Children looked for something in the chest of drawers”. **(F-H)** These images show the living environment post intervention. The clothes on hangers were removed **(F)**, the dolls in the bedroom were hidden using blindfold shades **(G)**, and the number of dolls in the living room was reduced **(H)**.

**Table 2** shows the clinical features of Patient 2. At admission, the patient’s CDR was 2. The MMSE and ACE-III scores were 5/30 and 33/100, respectively, indicating severe general cognitive impairment. The SFQ score was 6/8, indicating cognitive fluctuation. The MDS-UPDRS Part III score was 14/136, indicating postural instability due to muscle weakness. The total NPI score was 36/144, and the hallucination frequency and severity scores were 4/4 and 2/3, respectively. Caregiver distress for VH in the NPI-12 was severe. The mean ZBI score was 51/88. Cranial MR revealed bilateral atrophy of the medial temporal lobe and extensive high-intensity areas in the periventricular region and deep white matter (**Figure 2A**). ^123^I-IMP SPECT revealed an extensive reduction of brain perfusion (**Figure 2B**). Indicative biomarkers for DLB diagnosis were not assessed because of the patient’s strong refusal. Collectively, the patient was diagnosed with probable DLB according to the international diagnostic criteria (12).

**Table 2:** Clinical characteristics of Patient 2 at baseline and at 3-month follow up.

	Baseline			3 months follow up		
CDR	2			–		
MMSE	10/30 (pre-discharge)			14/30		
ACE-III	33/100			–		
SFQ	6/8			–		
MDS-UPDRS partIII	14/136			–		
PSMS	3/6			1/6		
NPI-12 score	Total score 36/144			Total score 17/144		
	Del 12/12	Hal 8/12	Agit 3/12	Del 3/12	Hal 4/12	Agit −
	Dep 3/12	Anx −	Eup −	Dep 2/12	Anx 2/12	Eup −
	Ap 4/12	DI 3/12	Irrit −	Ap 2/12	DI −	Irrit −
	MD −	NB 3/12	EA −	MD −	NB −	EA −
ZBI	51/88			43/88		
Visual hallucination						
Caregiver burden score (NPI-12 hallucination)	4/5 (severe)			0/5 (no burden)		
Frequency	Multiple times daily			Multiple times daily		
What the patient saw	Some foreign male strangers, children, dogs, cats			Animals such as cats or snakes		
When the symptoms occurred	Evening to night-time			Unspecified		
Visual features associated with hallucinations	Clothing on hungers in the bedroom (**Figure 2C**) Dolls in the living room and bedroom (**Figures 2D, E**)			Code of consumer electronics in the Kitchen		
How the patient reacted to the hallucinations	Believing that some male strangers intruded into the home and stole money			Not bothering with the visual hallucinations since she doesn’t hate those animals		

CDR, clinical dementia rating; MMSE, Mini-Mental State Examination; ACE-III, Addenbrooke’s Cognitive Examination III; SFQ, Short Fluctuations Questionnaire; MDS-UPDRS Part III, Movement Disorder Society Unified Parkinson’s Disease Rating Scale Part III; PSMS, Physical Self-Maintenance Scale; NPI-12, Neuropsychiatry Inventory-12; Del, delusions; Hal, hallucinations; Agit, agitation/aggression; Dep, depression/dysphoria; Anx, anxiety; Eup, elation/euphoria; Ap, apathy/indifference; DI, disinhibition; Irrit, irritability; MD, motor disturbance: NB, night-time behaviors; EA, appetite and eating abnormalities; ZBI, Zarit Burden Inventory.

The features of her VH were assessed using interviews and the PA-LE (**Table 2**). Interviews with family caregivers revealed that she frequently experienced seeing strangers at home. She also occasionally observed VHs of children and animals. The VH of the strangers was linked to the delusion of theft. She experienced VH several times daily in the living room and bedroom (**Figures 2C–E**). VH mainly occurred in the evening and at night. Based on the PA-LE, we assessed whether her VH experience had any possible environmental triggers. For instance, clothing on hangers in the bedroom, and dolls in the living room and bedroom could be inducing her VH experiences, although we could not assess whether the VH occurred every time she saw the triggers. The patient described “Male strangers intruded my home from the balcony,” and “Children looked for something in the chest of drawers.” Her daughter did not know how to deal with the VH, delusions, and aggression, and was confused.

Donepezil hydrochloride (3 mg/day) was prescribed during hospitalization, with a gradual increase to 5 mg/day. The suvorexant dosage was also increased to 15 mg/day for insomnia. The occurrence of VH and delusions gradually decreased, and aggression and irritability improved. The MMSE score increased to 10/30. Although the donepezil hydrochloride dosage was reduced to 3 mg/day due to a reduction in appetite and nausea, the improvement was maintained. For further management of VH after discharge, we made several recommendations to the patient and her daughter (**Supplementary Table**). First, we provided them with environmental adjustments, for example, to remove or hide clothes hanging on walls and dolls, which were considered environmental triggers of VH. Second, we encouraged them to spend time together doing household activities or going out and engaging in leisure activities such as handcrafts, to reduce general anxiety. Finally, we suggested a coping strategy that suggested that caregivers respond to the patient for a feeling of security if a VH occurred.

The patient had an uneventful 3-month follow-up. Her daughter implemented the provided guidance, and the environmental triggers were reduced (**Figures 2F–H**). To increase the pleasure in the patient’s daily life, the patient’s daughter went shopping with her once a week, and she began to enjoy the leisure activity of origami. The NPI score improved not only in the hallucination subdomain but also in the delusion or agitation/aggression subdomains (**Table 2**). Caregiver distress in terms of the VH decreased to zero. The daughter’s knowledge of VH was altered as follows: “I discovered that the invisible people might be induced by environmental stimuli.” The general caregiver burden, as assessed by the ZBI, also decreased to 43/88. Regarding VH content, the male strangers disappeared, although the VH of the animals persisted. Delusions associated with the male strangers also improved.

## 3 Discussion

This report describes two patients with DLB whose VH was evaluated through phenomenological assessments and photographs of their homes. We revealed the potential association between environmental factors and the occurrence of VH. To the best of our knowledge, this is the first case report to describe in detail the method of environmental photo assessment and adjustment for improving VH. This approach is likely to be useful not only for improving VH related to environmental stimuli, but also for reducing caregiver burden by enhancing caregivers’ knowledge of VH.

A combination of disturbed visuospatial processes and impaired attentional binding has been suggested to play a key role in the development of VH in DLB (13). VH and visual illusion are thought to be similar and closely related in DLB, although they have different symptoms according to their definitions (6). In the present cases, it is conceivable that environmental adjustment to remove the triggers of the visual illusion would likely contribute to the improvement of the patient’s VH. Importantly, specific comments such as “Someone is in my bedroom” in Patient 1 and “Male strangers intruded my home from the balcony” in Patient 2 disappeared after the intervention. However, several VHs persisted or began in both patients after environmental adjustments. Patient 1 reported “visitors are around a wall in the living room” and Patient 2 reported “a snake is near the electronics in the kitchen.” This outcome supports the potential explanation that our method may be useful for decreasing environmental triggers of visual illusions and partially modifying the VH experience. Our findings are in line with a previous study that found that the improvement of VH was accompanied by an improvement in visual illusions in patients with DLB who responded to cholinergic enhancement treatment (6).

This intervention also enhanced caregivers’ knowledge of the characteristics and coping methods of VH, leading to a decrease in caregiver burden. We provided them with individualized information about environmental triggers and care plans for VH using relevant photographs of their patients’ homes. In a previous study, a better knowledge of care methods or symptoms was shown to affect caregivers’ attitudes toward patients (14). Regarding caregiver education, an individualized program is more effective than the usual care plan in managing problem behaviors and increasing confidence (15), and visual tools have been suggested to facilitate a better understanding (16). Several factors, such as individualized and visual content, might increase caregivers’ knowledge of VH and care methods, leading to a reduced caregiver burden. Furthermore, caregivers could detect environmental triggers by preparing photographs of their patients’ homes themselves. This active experience also seems to promote the acquisition of knowledge (17). The general care burden assessed using the ZBI increased after the intervention in Patient 1, although the patient’s VH improved. It is important to note the possible negative effect that our intervention might have led to some degree of stressful conflict for the caregivers. However, this would be explained by the worsening of agitation/aggression symptoms in daily care routines or an increase in the care of her husband, considering the burden score of each subitem score in NPI and clinical course in Patient 1.

This case report had some limitations. First, as this approach consists of several components, it would be difficult to determine the pure effect of environmental adjustment on VH. Its effect could not be clearly distinguished from that of pharmacological interventions, especially in Patient 2. Secondly, co-pathologies have not been confirmed in detail in these patients, although psychosis has been reported to be involved in various neuropathologies (18, 19).

In conclusion, phenomenological assessment using photographs of the patient’s home assisted in identifying the environmental factors associated with VH in patients with DLB. PA-LE is useful for the assessment and intervention of VH partially associated with visual prediction error.

Further prospective studies with larger populations are required to reveal common patterns of environmental triggers that induce VH. Assessment of the changes in VH during step-by-step environmental adjustments could verify the effect of removing triggers on VH.

## Data availability statement

The datasets presented in this article are not readily available because The datasets generated during the current report are not publicly available due to privacy considerations of the participants. Requests to access the datasets should be directed to Hideki Kanemoto,hkanemoto@psy.med.osaka-u.ac.jp.

## Ethics statement

Written informed consent was obtained from the individual(s) and their families for the publication of any potentially identifiable images or data included in this article.

## Author contributions

DI: Conceptualization, Project administration, Writing – original draft. HK: Conceptualization, Project administration, Writing – review & editing. MH: Writing – review & editing. YN: Writing – review & editing. FK: Project administration, Writing – review & editing. YS: Project administration, Writing – review & editing. DT: Project administration, Writing – review & editing. MI: Supervision, Writing – review & editing.

## References

[1] Eversfield CL Orton LD. Auditory and visual hallucination prevalence in Parkinson’s disease and dementia with Lewy bodies: a systematic review and meta-analysis. Psychol Med. (2019) 49:2342–53. doi: 10.1017/s0033291718003161 PMC6763539 30474581

[2] Tzeng R-C Tsai C-F Wang C-T Wang T-Y Chiu P-Y. Delusions in patients with dementia with Lewy bodies and the associated factors. Behav Neurol. (2018) 2018:6707291. doi: 10.1155/2018/6707291 29854018 PMC5964573

[3] Kanemoto H Sato S Satake Y Koizumi F Taomoto D Kanda A. Impact of behavioral and psychological symptoms on caregiver burden in patients with dementia with Lewy bodies. Front Psychiatry. (2021) 12:753864. doi: 10.3389/fpsyt.2021.753864 34777057 PMC8578553

[4] Mosimann UP Rowan EN Partington CE Collerton D Littlewood E O’Brien JT. Characteristics of visual hallucinations in Parkinson Disease dementia and dementia with Lewy bodies. Am J Geriatric Psychiatry. (2006) 14:153–60. doi: 10.1097/01.jgp.0000192480.89813.80 16473980

[5] Collerton D Barnes J Diederich NJ Dudley R Friston K Goetz CG. Understanding visual hallucinations: A new synthesis. Neurosci Biobehav Rev. (2023) 150:105208. doi: 10.1016/j.neubiorev.2023.105208 37141962

[6] Yokoi K Nishio Y Uchiyama M Shimomura T Iizuka O Mori E. Hallucinators find meaning in noises: Pareidolic illusions in dementia with Lewy bodies. Neuropsychologia. (2014) 56:245–54. doi: 10.1016/j.neuropsychologia.2014.01.017 24491313

[7] O’Brien J Taylor JP Ballard C Barker RA Bradley C Burns A. Visual hallucinations in neurological and ophthalmological disease: pathophysiology and management. J Neurol Neurosurg Psychiatry. (2020) 91:512. doi: 10.1136/jnnp-2019-322702 32213570 PMC7231441

[8] Yumoto A Suwa S. Difficulties and associated coping methods regarding visual hallucinations caused by dementia with Lewy bodies. Dementia. (2021) 20:291–307. doi: 10.1177/1471301219879541 31610695

[9] Connors MH Quinto L McKeith I Brodaty H Allan L Bamford C. Non-pharmacological interventions for Lewy body dementia: a systematic review. Psychol Med. (2018) 48:1749–58. doi: 10.1017/s0033291717003257 PMC6088773 29143692

[10] Ishimaru D Kanemoto H Hotta M Nagata Y Satake Y Taomoto D. Case report: Treatment of delusions of theft based on the assessment of photos of patients’ homes. Front Psychiatry. (2022) 13:825710. doi: 10.3389/fpsyt.2022.825710 35370805 PMC8968168

[11] McKeith IG Dickson DW Lowe J Emre M O'Brien JT Feldman H. Diagnosis and management of dementia with Lewy bodies: Third report of the DLB consortium. Neurology. (2005) 65:1992. doi: 10.1212/wnl.65.12.1992-a 16237129

[12] McKeith IG Boeve BF Dickson DW Halliday G Taylor J-P Weintraub D. Diagnosis and management of dementia with Lewy bodies: Fourth consensus report of the DLB Consortium. Neurology. (2017) 89:88–100. doi: 10.1212/wnl.0000000000004058 28592453 PMC5496518

[13] Collerton D Perry E McKeith I. Why people see things that are not there: A novel perception and attention deficit model for recurrent complex visual hallucinations. Behav Brain Sci. (2005) 28:737–57. doi: 10.1017/S0140525X05000130 16372931

[14] Teichmann B Gkioka M Kruse A Tsolaki M. Informal caregivers’ attitude toward dementia: The impact of dementia knowledge, confidence in dementia care, and the behavioral and psychological symptoms of the person with dementia. A cross-sectional study. J Alzheimer’s Dis. (2022) 88:971–84. doi: 10.3233/jad-215731 PMC9484115 35723101

[15] Gitlin LN Winter L Dennis MP Hodgson N Hauck WW. Targeting and managing behavioral symptoms in individuals with dementia: A randomized trial of a nonpharmacological intervention. J Am Geriatr Soc. (2010) 58:1465–74. doi: 10.1111/j.1532-5415.2010.02971.x PMC2955191 20662955

[16] Udow SJ Hobson DE Kleiner G Masellis M Fox SH Lang AE. Educational needs and considerations for a visual educational tool to discuss Parkinson’s disease. Mov Disord Clin Pract. (2017) 5:66–74. doi: 10.1002/mdc3.12563 30363445 PMC6174478

[17] Bobek E Tversky B. Creating visual explanations improves learning. Cogn Res Princ Implic. (2016) 1:27. doi: 10.1186/s41235-016-0031-6 28180178 PMC5256450

[18] Satake Y Kanemoto H Taomoto D Suehiro T Koizumi F Sato S. Characteristics of very late-onset schizophrenia-like psychosis classified with the biomarkers for Alzheimer’s disease: a retrospective cross-sectional study. Int Psychogeriatr. (2024) 36, 64–77. doi: 10.1017/s1041610222001132 36714996

[19] Kanemoto H Satake Y Suehiro T Taomoto D Koizumi F Sato S. Characteristics of very late-onset schizophrenia-like psychosis as prodromal dementia with Lewy bodies: a cross-sectional study. Alzheimers Res Ther. (2022) 14:137. doi: 10.1186/s13195-022-01080-x 36138485 PMC9503193

