# Supplementary figures and images for: Case report: Environmental adjustment for visual hallucinations in dementia with Lewy bodies based on photo assessment of the living environment
Source: Front Psychiatry. 2024 Mar 15;15:1283156. doi: 10.3389/fpsyt.2024.1283156 (PMC10978580; doi:10.3389/fpsyt.2024.1283156)

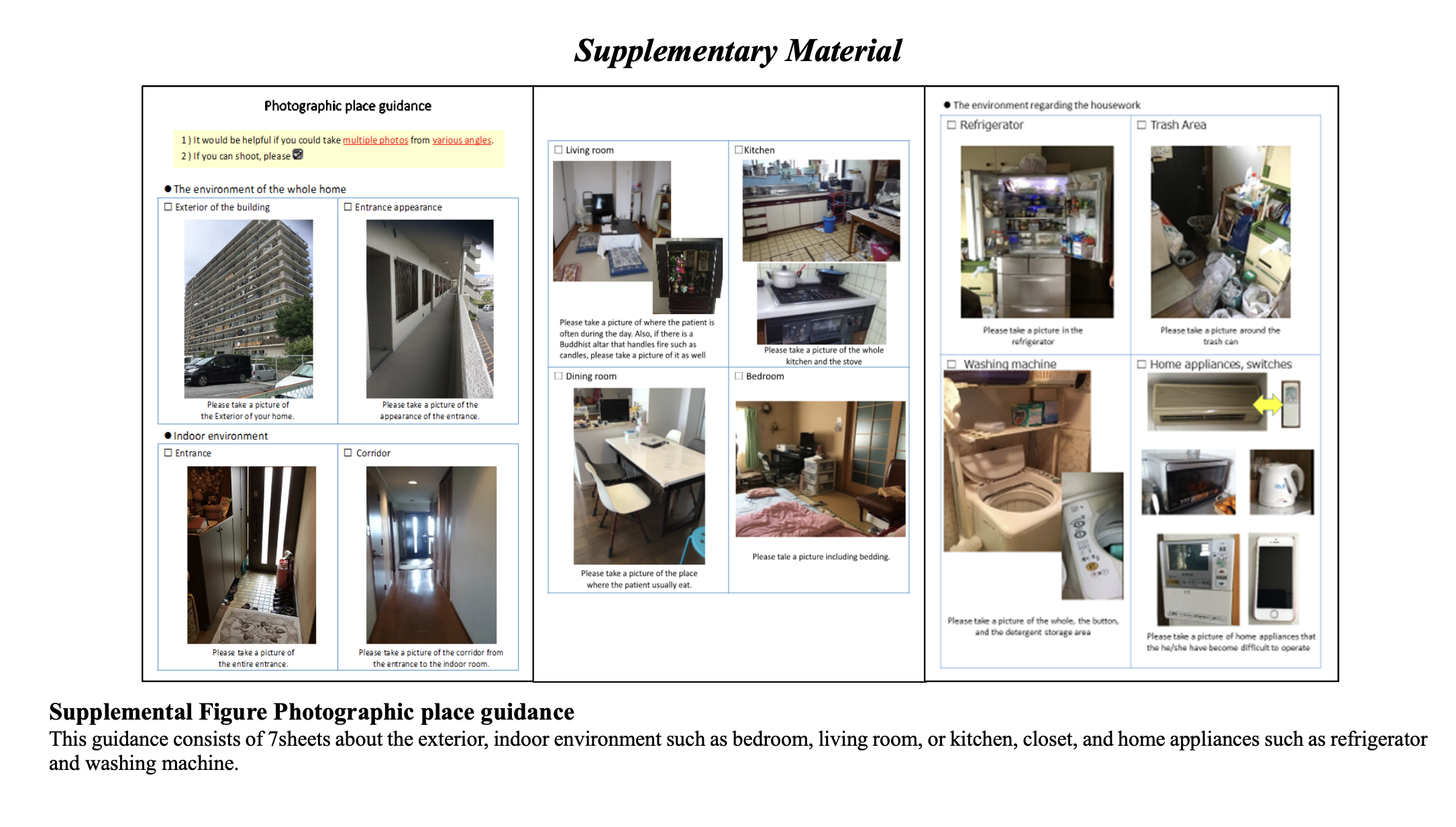

Supplement: Supplementary file 1 [file Image_1.tiff]
