# Supplementary material for: Case report: Environmental adjustment for visual hallucinations in dementia with Lewy bodies based on photo assessment of the living environment
Source: Front Psychiatry. 2024 Mar 15;15:1283156. doi: 10.3389/fpsyt.2024.1283156 (PMC10978580; doi:10.3389/fpsyt.2024.1283156)
Supplement: Supplementary file 2 [file Table_1.docx]

Supplementary Material

**Supplemental Table Content of the non-pharmacological intervention for visual hallucinations**

| Case 1 | Case 2 |
| --- | --- |
| 1. **Environmental adjustment**   Removing or hiding the environmental triggers associated with visual hallucinations was advised.  - Putting away cushions on a sofa in the evening and at night  - Hiding the pattern on the carpet under the table using a tablecloth  - Folding clothes rather than putting them on hangers in the bedroom or storing the clothes if putting them on hangers | 1. **Environmental adjustment**   Removing or hiding the environmental triggers associated with visual hallucinations was advised.  - Folding clothes rather than putting them on hangers in the bedroom or storing the clothes if putting them on hangers  - Hiding the dolls using blindfold shades and reducing the number of dolls |
| 1. **(b) Reducing general anxiety**   The husband was encouraged to increase pleasure in the patient’s daily life. This included items such as attending an adult day care or spending time together, particularly in the evening and at night. | **(b) Reducing general anxiety**  The daughter was encouraged to increase pleasure in the patient’s daily life. This included items such as doing households chores together, going out together, and engaging in leisure handcrafting activities. |
| **(c) Providing a coping strategy for when the visual hallucinations occurred**  The coping strategy involved the patient touching something that only she could see if visual hallucinations occurred. We encouraged her husband to respond to her for a feeling of security. | **(c) Providing a coping strategy for when the visual hallucinations occurred**  The coping strategy involved caregivers responding to the patient for a feeling of security when she reported visual hallucinations. The coping strategy involving touching something that only the patient could see was not suggested since the patient trended not to experience visual hallucinations in real time. |
